# Linking Production and Consumption: The Role for Fish and Seafood in a Healthy and Sustainable Australian Diet

**DOI:** 10.3390/nu11081766

**Published:** 2019-08-01

**Authors:** Jessica R. Bogard, Anna K. Farmery, Danielle L. Baird, Gilly A. Hendrie, Shijie Zhou

**Affiliations:** 1 CSIRO, Agriculture and Food, Brisbane 4067, Australia; 2 University of Wollongong, Australian National Centre for Ocean Resources and Security, Wollongong 2522, Australia; 3 CSIRO, Health and Biosecurity, Adelaide 5000, Australia; 4 CSIRO, Oceans and Atmosphere, Brisbane 4072, Australia

**Keywords:** Fish, seafood, food system, nutrition, diet, sustainability, Australia, sustainable diet

## Abstract

Fish and seafood consumption in Australia has been growing, yet the implications of this trend across the food system, from both a health and sustainability perspective have not been fully explored. This paper aims to map out the fish and seafood food system in Australia, linking production and consumption, to articulate challenges and opportunities for enhancing the sector’s contribution to future healthy and sustainable diets. We conducted a secondary analysis of publicly available datasets on fish and seafood production and consumption, triangulated and supplemented with peer-reviewed and grey literature on environmental, economic and social sustainability issues throughout the food system. A key challenge for health is the high proportion of fish and seafood consumed as discretionary food, particularly among children. Key challenges for sustainability include the narrow focus on environmental sustainability (with little consideration of the other domains), and the focus on production with little consideration for sustainability throughout post-harvest handling, processing, retail, distribution and consumption. Key opportunities for health and sustainability include the innovative use of processing and packaging technology to maximise nutritional quality; creation of markets and supply chains for a greater diversity of underutilised fish and seafood species and processing by-products; and reductions in waste and loss throughout the entire supply chain.

## 1. Introduction

The global food system has never been more advanced, and yet is also considered a key driver of malnutrition in all its forms, as well as unprecedented environmental damage including land degradation, greenhouse gas emissions, water use, rapid deforestation and biodiversity loss [1,2]. The adoption of healthy and sustainable diets and food systems is, therefore, increasingly recognised as a key strategy in addressing this dual challenge and achieving the Sustainable Development Goals (SDGs) [3,4,5]. A major challenge, however, relates to the inherent complexity of food systems. A food system has been defined as “all the elements (environment, people, inputs, processes, infrastructures, institutions, etc.) and activities that relate to the production, processing, distribution, preparation and consumption of food, and the output of these activities, including socio-economic and environmental outcomes” [4]. Interventions in food systems tend to be sector specific, focusing on a single element or sector within the system without broad consideration for how changes in one area will inevitably trigger actions and reactions throughout the system. A ‘whole-of-food-system’ approach that specifically considers these interactions is essential for optimising food system outcomes for both health and sustainability.

The Australian Dietary Guidelines (ADGs) recommend the consumption of 140–280 g of fish per week for adults to achieve health benefits including reduced risk of cardiovascular disease, stroke, dementia and macular degeneration [6]. Health outcomes from fish consumption, in addition to total quantity, also depend on the species, the way in which fish and seafood products are processed and prepared, and the other ingredients or foods consumed as part of a meal. It is, therefore, essential that health recommendations be based on high-quality food consumption data and underpinned by food composition data that reflects the appropriate variation in species, preparation methods and other food components for the given population [7]. Total per-capita seafood consumption in Australia increased by 45% between 1995 and 2011/12, driven by increases in both the *quantity* consumed by seafood consumers (in other words, the portion size consumed) and the *proportion* of seafood consumers as a percentage of the total population [8]. The largest increases have been in canned and other processed seafood categories. The quantity of canned fish and processed fish per capita increased by 111 and 89% respectively; whilst the proportion of consumers of canned fish and processed fish (as a % of total population) increased by 77 and 100% respectively. The implications of this growing trend across the food system, from both a health and sustainability perspective have not been explored. Doing so requires an understanding of the entire fish and seafood food system in Australia including the major sources of supply and the transformation of fish and seafood products that occurs throughout the supply chain to the point of consumption, including waste and loss. 

The sustainability of fish production systems and products has received intense scrutiny in recent years (see, e.g., [9,10,11]). While sustainability broadly encompasses three dimensions—environmental, economic and social [12]—the social and economic aspects of seafood sustainability have been largely underrepresented [13]. In Australia, this may be in part due to the fact that there are substantive policy gaps in regard to social and economic goals for Australia’s fisheries management [14]. Globally, the environmental sustainability of fish production systems are regulated through national laws and local regulations, as well as through regional and international bodies, including Regional Fishery Management Authorities, and agreements, such as the Code of Conduct for Responsible Fisheries [15]. The enforcement of these national and international instruments varies substantially [16], thereby influencing the sustainability of production. For most wild capture fisheries, ‘sustainability’ is formally assessed through government lead stock assessments, the results of which are incorporated into the bi-annual State of World Fisheries and Aquaculture reports [17]. The focus of sustainability in fisheries is on the harvesting rate of the target stock to ensure the population does not decline over time. An ecosystem-based fisheries management (EBFM) approach has been promoted as an alternative to traditional fisheries management [18], and adopted by Australian fisheries managers. However, ecosystem structure and biological diversity are not currently assessed as part of formal sustainability assessments. 

Market-oriented initiatives have also emerged to provide consumers with an assessment of seafood sustainability. Sustainable seafood guides, seafood sourcing policies, voluntary labelling guidelines, and third-party certification schemes are examples of non-state driven governance tools [19]. Seafood guides, such as those developed by the Monterey Bay Aquarium (seafoodwatch.org) and the Australian Marine Conservation Society (AMCS, sustainableseafood.org.au), incorporate government stock assessment data with their own assessment of broader issues including ecological considerations such as bycatch and the impact of fishing on habitats. Certification bodies such as the Marine Stewardship Council (MSC, msc.org) and the Aquaculture Stewardship Council (ASC, asc-aqua.org) have developed scoring frameworks with performance indicators that need to be met for certification. 

In addition, life cycle assessment (LCA) has been used to assess and compare seafood supply chains and provide new insights into the environmental impact of seafood products [20]. An important consideration for seafood sustainability identified through LCAs is the target species and stock status, as schooling and abundant species can be harvested more efficiently (see, e.g., [21,22]). In addition, the type of fishing gear used is an important aspect of sustainability (see, e.g., [23,24]) as is the amount of fuel used [25]. For aquaculture, the type and amount of feed is an important consideration, given the production of feed ingredients often dominates LCA results (see, e.g., [26,27]). In addition to the sustainability of production, the product yield and post-harvest losses can influence the sustainability of products [21] as can the mode of transport and energy source used for processing [28,29]. Seafood LCAs have predominantly addressed global-scale abiotic resource use and emission-based environmental impacts, such as global warming, toxicity and eutrophication [20]. Efforts are being made to include more biological impacts of fishing (see, e.g., [30,31]), as well as social and economic aspects of sustainability (see, e.g., [32,33,34]). LCAs are also evolving to measure impacts in relation to a broader range of functions than product weight or value, for example Hallström et al. [35] recently linked seafood sustainability to nutritional content of products.

Addressing food losses and waste has been recognised as a key component of improving the sustainability of food systems [1], and reducing food loss and waste by half is a specific target of the SDGs (target 12.3). The Australian Government has committed to this target through the National Food Waste Strategy launched in 2017 [36]. Under this strategy, the National Food Waste Baseline Assessment report estimated that in 2016/17, Australia produced 7.3 million tonnes (t) of food waste; 31% at the primary production stage, 25% in the manufacturing sector and 31% at the household level [37]. This baseline assessment did not provide an estimate of seafood waste in the primary production stage; though estimated 50,080 t of seafood waste in the manufacturing stage, and 11,400 t in the wholesaling stage. However, the report acknowledges that confidence in the data is low due to limitations in the methods. Better information on the quantity of waste and loss occurring throughout the fish and seafood supply chain, and the driving forces behind this waste and loss is crucial for improving sustainability and meeting the SDG target.

This paper aims to map out the fish and seafood food system in Australia, linking production and consumption (both overall, and of specific products) to articulate challenges and opportunities for enhancing its contribution to future healthy and sustainable diets. We find that sustainability assessment frameworks currently used in Australia are narrowly focused on environmental aspects of production and fail to consider the economic and social aspects of sustainability, as well as sustainability throughout the remaining stages of the food supply chain. Key opportunities for increasing the contribution of fish and seafood to health and sustainability goals include the innovative use of processing and packaging technology to maximise nutritional quality; creation of markets and supply chains for a greater diversity of underutilised fish and seafood species to accommodate increasing demand from consumption; and reductions in waste and loss throughout the entire supply chain.

## 2. Materials and Methods 

We conducted a secondary analysis of publicly available datasets on fish and seafood production and consumption, triangulated and supplemented with peer-reviewed and grey literature on relevant sustainability issues including food loss and waste, to map the flow of fish and seafood products through the food system in Australia. We use the phrase ‘fish and seafood’ throughout to be consistent with the Australian Dietary Guidelines [6]. The term ‘fish’ is used to refer to finfish of saltwater and freshwater origin. The term ‘seafood’ is used to refer collectively to saltwater and freshwater fish, molluscs, cephalopods, and crustaceans. The details of the secondary analysis are described below. Data related to processing, distribution and retail of fish and seafood products is largely held by the private sector and so there is limited publicly available data to map out these dimensions. In this case, information from published and grey literature were sought to provide some insights. 

### 2.1. Production

Publicly available production statistics for capture fisheries and aquaculture, including details of imports and exports were obtained from the Australian Bureau of Agricultural and Resource Economics and Sciences (ABARES) division of the Commonwealth Department of Agriculture and Water Resources [38]. ABARES collates data on fishing catch provided by state and territory fisheries departments and the Australian Fisheries Management Authority for Commonwealth managed fisheries. ABARES also acknowledge input from researchers and industry representatives in collating fisheries and aquaculture data [39]. Fishing catch data for fisheries managed by quotas (fisheries where a total allowable catch limit has been set for commercial fishers) is collected through the use of Catch Disposal Records (CDRs). On landing, fisher licence holders are required to complete a CDR detailing the species and quantity of fishing catch, in addition to other information. When a CDR is not completed, fisher logbooks are used to determine catch levels which are collated by state and territory departments and provided to ABARES. ABARES also collates data on imports and exports of fisheries products provided to them by the Australian Bureau of Statistics (ABS). The ABARES data was triangulated with data from the Food and Agriculture Organization (FAO) Global Fishery and Aquaculture Commodities Statistics database (v2018.1.0) and the FAO food balance sheets of fish and fishery products (v2017.1.0), both analysed using FishStatJ (release 3.5.0). Data from December 2011 was used to provide consistency with the availability of population consumption data described below.

### 2.2. Consumption

Consumption data on fish and seafood products was obtained from a secondary analysis of dietary intake data collected in the 2011–12 National Nutrition and Physical Activity Survey as part of the Australian Health Survey (AHS) conducted by the ABS. This survey collected dietary intake data from a nationally representative sample of 12,153 participants aged 2 years and over and is the most recent and comprehensive data available on food consumption patterns in Australia (for detailed information on the sampling design see [40]). More recent estimates of the total amount of seafood consumed in Australia have been calculated by adding the total edible quantity of seafood supplied domestically (domestic production plus imports), less exports of seafood. This data is useful for tracking national consumption patterns but is less comprehensive than the AHS data as it does not include information on sociodemographic traits of consumers, or on species of seafood consumed by individuals. Using the ABS food classification system, foods were flagged and identified as a fish/seafood product for the purpose of this analysis if the content of fish or seafood was greater than 1%. This only excluded aquatic plants such as seaweed and condiments such as fish sauce. 

Dietary intake data was collected via 24-h recall on two separate days using an Automated Multiple-Pass Method. All foods and beverages consumed on the day prior to the interview (midnight to midnight) were recalled, including time of consumption, eating occasion, detailed food description and amount eaten. The secondary analysis conducted for this paper utilised data collected on the first day of recall. Weighting factors were applied to summary estimates to reflect the demographic structure of the Australian population (based on age, gender and residential area). For inferential statistics, the population weights were rescaled to the size of the sample. In addition, due to a disproportionate number of recalls occurring by day of week (under representation of Sundays and to a lesser extent Fridays), an additional weighting factor was applied to correct for the day of the week the survey was recorded. 

The methods used here build on a recent sociodemographic analysis of fish consumption patterns in Australia [41] in three important ways. Firstly, we adjust for potential underreporting of total energy intake by participants, which is common in all nutrition surveys and is important when trying to estimate the true impact of seafood consumption throughout the food system. The ABS has produced estimates of the degree of underreporting in this survey, suggesting a correction factor of 1.21 for females and 1.17 for males to adjust for the misreporting of energy. While misreporting is likely to vary by weight status and food type, this level of correction has not been provided for these data. Therefore, the correction factors were applied uniformly to all food intake data. Secondly, for mixed dishes containing seafood as a major component, we disaggregated recipes to identify the seafood components within the dish and only included this component for analysis. For example, in a tuna mornay recipe, only the tuna component of this recipe contributed to seafood estimates, not the sauce, pasta or vegetables. Thirdly, we also include analysis of the seafood component of mixed dishes that include seafood as a minor component, for example, the seafood component of a seafood pizza.

To better enable the interpretation of consumption in relation to production and supply, seafood categories from within the dietary survey data are presented according to three different categorisation structures. First by seafood type; ‘fish’ (salmon, tuna, barramundi, sardines or other fish species), ‘crustaceans’ (prawns or other crustaceans), ‘molluscs’ (oysters or other molluscs) and ‘other seafood’ (unspecified/mixed seafood). Second, by level of processing of the products based on the description used in the dietary data; ‘fresh or frozen’ (otherwise unprocessed), ‘canned’, ‘smoked’, ‘other processing’ (crumbed/battered and other processed products) and ‘ingredient in mixed dish’. Finally, fish and seafood products were classified as a core or discretionary food consistent with the Australian Dietary Guidelines [6] and flagged in the dataset by the ABS [40]. In the context of fish and seafood, discretionary food included commercially fried foods, and coated or crumbed products that included more than 5 g of saturated fat per 100 g. Of the 391 foods identified as seafood or seafood containing, 89 (22.7%) were discretionary. Examples include, fish cakes, crumbed squid/calamari, fish fingers, coated fish products and seafood/fish stick (surimi).

Survey participants were scored and ranked according to their diet quality using a food-based Dietary Guideline Index [42]. The Diet Quality Index (DQI) assesses whole-of-diet quality as overall compliance with the ADGs, assessing quality, quantity and variety of food and beverages consumed. The Index is scored out of 100, where a higher score reflects greater compliance with dietary guidelines. Participants were ranked into tertiles of diet quality, enabling comparison of seafood consumption across tertiles of diet quality. 

Statistical analyses were performed using the IBM SPSS statistical software package version 23 (SPSS Inc., Chicago, IL, USA). Fish consumption was examined by aggregated groups of children aged 2–18 years and adults aged 19+ years and by tertiles of diet quality. Estimates were calculated for the proportion of the population group who consumed seafood on the day surveyed, and for each fish classification (fish type, processing type and core/non-core) the mean (SD) daily intake was estimated for the whole population as well as for consumers of any seafood. 

Differences in mean fish consumption between tertiles of diet quality was tested for statistical significance using one-way ANOVA with post-hoc comparison. Chi Square analysis was used to test for differences in the proportion of seafood consumers by tertiles of diet quality. Weighting factors were rescaled to the size of the sample for inferential statistics and *p*-values of less than 0.01 were taken as indicating statistical significance.

### 2.3. Sustainability

Sustainability is a complex concept encompassing many different elements, metrics and methods of assessment. We do not calculate specific measures of sustainability here, instead we interpret sustainability in the context of the three dimensions—environmental, economic and social—at every stage of the fish and seafood food system and use published information to highlight specific challenges and opportunities for sustainability. Key considerations for seafood sustainability relevant at each stage of the supply chain were identified from the seafood literature [20], and the environmental, social and economic principles for achieving a sustainable food system identified by the United Nations High-Level Taskforce for Food and Nutrition Security (HLTF) [43]. These key considerations were used to generate a matrix comparing the extent of consideration of these issues among the four main seafood sustainability assessment guides used in Australia; the Australian Government framework applied to assessment of capture fisheries [44], the MSC’s Principles and Criteria for Sustainable Fishing [45], the ASC’s standards for aquaculture [46], and the AMCS’s Sustainable Seafood Guide [47]. 

A comprehensive assessment of waste and loss throughout the Australian fish and seafood food system is lacking. We, therefore, estimate waste and loss here by applying waste and loss coefficients for fish and seafood according to quantity of products at each stage of the food system, from an FAO global study with specific estimates for the North America and Oceania region (based on literature from Australia, Canada, New Zealand and USA) [48]. Waste and loss refer to the decrease in edible food mass throughout the supply chain and does not consider parts of products which are not edible. Coefficients used were: 12% at production (referring to discards), 0.5% at post-harvest handling and storage (spillage and degradation during icing, packaging, storage and transportation after landing), 6% at processing and packaging (industrial processing such as canning and smoking), 9% at distribution and retail (wholesale markets, supermarkets, retailers and wet markets), and 15% at consumption (at the household level).

## 3. Results

### 3.1. Production, Imports, Exports and Processing

**Figure 1:**
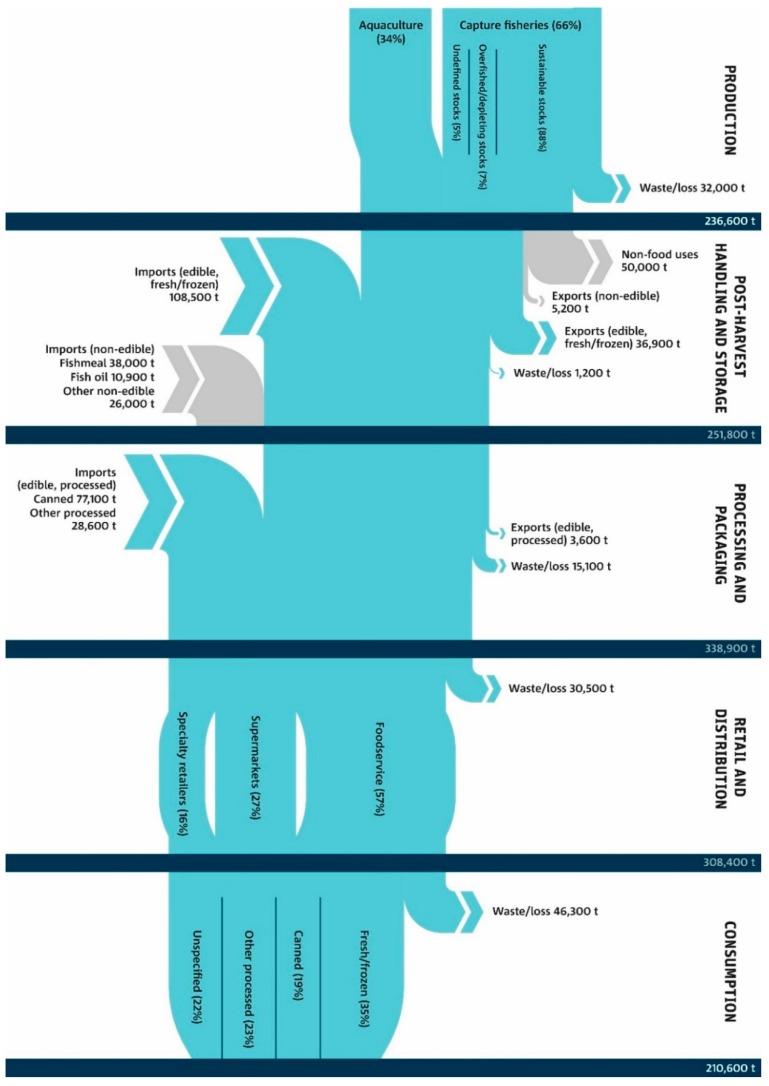
Flow of fish and seafood from production to consumption in Australia in December 2011.

The flow of fish and seafood from production through post-harvest handling and storage, processing and packaging, retail and distribution, and consumption in Australia is shown in Figure 1. In 2011–2012, Australia produced approximately 236,600 t of fish and seafood; approximately 66% was from capture fisheries and the remaining 34% from aquaculture. Of total domestic production, approximately 21% (50,000 t) was exported (as per FAO Fishstat data); mostly as edible seafood (40,500 t), the vast majority of which was fresh or frozen (91%) and the remaining 9% was processed prior to exporting. The quantity of non-edible exports is not reported in ABARES data, but includes marine fats and oils, fish meal, pearls and ornamental fish with an economic value of AUD 226 million (18% of total fisheries exports); though the quantity of non-edible exports according to FAO data was 5200 t. An additional 210,000 t of edible seafood was imported; 51% was fresh or frozen seafood, 36% was canned (mostly tuna), and 13% was as other forms of processed seafood such as smoked, dried or otherwise prepared seafood. According to FRDC analysis of ABS data, less than 1% of imported edible seafood is from re-imports (seafood produced in Australia then exported for processing before being re-imported) [49]. Similar to non-edible exports, the quantity of non-edible imports is not specified in ABARES data but amounts to approximately AUD 233 million (14% of the total value of fisheries imports). According to FAO Fishstat data; approximately 38,000 t of fish meal, 11,000 t of fish oil and 26,000 t of other non-edible seafood was imported. According to FAO food balance sheets, 50,000 t of Australian seafood was directed to non-food uses. Details on non-food uses of seafood are not readily available in Australia. However, estimates from 2003 indicate that 33,600 t of seafood (47% of which was imported) was used for pet food [50]. The other major uses include reduction into fishmeal and fish oil as inputs for animal feed (including aquaculture as well as terrestrial animal production) and possibly fish oil supplements for human consumption. 

### 3.2. Retail and Distribution

After adjustments were made to total production quantities for imports, exports, non-food uses and waste, it is estimated that approximately 308,400 t of seafood is available for consumers to purchase or consume via retail or foodservice outlets. Of this, approximately 37% is sourced from domestic production and the remaining 63% is from imported seafood. This estimate is remarkably close to the 307,000 t of seafood distributed through domestic sales estimated in the Department of Agriculture Fisheries and Forestry FOODmap analysis for the same period [51]. According to that analysis, the primary distribution channel for seafood to consumers in Australia is via foodservice which includes takeaway outlets, restaurants, institutional foodservice (such as food provided through hospitals, prisons, schooling and child care facilities) and catering at special events [51]. Supermarkets account for approximately 27% of seafood retail quantities and specialty retailers such as fishmongers account for the remaining 16%. This indicates that consumers (excluding recreational fishers who acquire fish for consumption outside formal supply chains) are not directly involved in the cooking and preparation of the majority of seafood that is consumed.

Proportion of capture fisheries supply considered sustainable, depleting, overfished or undefined depicted here are as per Australian Government reporting framework based on abundance of fish stocks (biomass) and the level of fishing pressure [44]. An equivalent reporting framework for the sustainability of aquaculture products does not exist. However, domestic production systems must comply with national regulations, such as the Environment Protection and Biodiversity Conservation Act 1999. Numbers are rounded to the nearest 100 t.

### 3.3. Consumption Patterns

**Figure 2:**
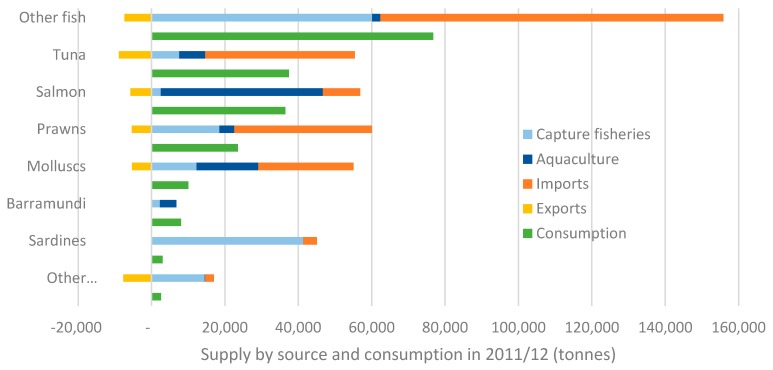
Australian consumption and supply of major fish and seafood types by production source in 2011/2012 (tonnes).

The total quantity of major seafood types consumed in Australia in December 2011 is shown in Figure 2 alongside the supply source. Tuna, salmon and prawns were the most popular types of seafood consumed (excluding the diverse category of ‘other fish’ which contains more than twenty different types of fish). The majority of tuna is supplied through imports; nearly all of which is canned tuna, of which 95% comes from Thailand (data not shown). The majority of salmon is supplied through domestic aquaculture. The majority of prawns in supply in Australia are supplied through imports; China, Thailand and Vietnam supply 34, 30 and 23% of total prawn imports, respectively (data not shown). 

In most cases, the large discrepancy between total domestic supply and consumption of seafood categories can be explained by the difference between whole live weight of seafood (the way in which supply is reported) and what is commonly considered as ‘edible parts’ (the way in which consumption is reported). For example, a whole live salmon is approximately 1.6 times the weight of the raw salmon steaks typically sold to consumers; a whole prawn with shell and tail is approximately 2.8 times the weight of raw prawn flesh typically consumed; mussels in shells are approximately 6 times the weight of raw mussel flesh consumed [52]. A notable exception to this discrepancy is the case of sardines, where it is known that nearly all domestic catch is diverted for use as an input to aquaculture feed [53], and consumption is largely consistent with the quantity of sardines imported. Waste and loss throughout the seafood supply chain will also account for some of the discrepancy. 

‘Other fish’ category consists of more than 20 different species such as basa, trevalla, cod, hake, flathead, bream, snapper, whiting, mullet. Figure excludes non-specified seafood supply and consumption.

Approximately 25% of adults, and 15% of children consumed fish or seafood on the day of the survey with a national average consumption of 31 and 12 g/person/day by adults and children respectively. Among seafood consumers, mean consumption was 123 and 80 g/person/day among adults and children respectively. The proportion of seafood consumers is higher than previously reported [41] given that we include analysis of those consumers where seafood was a minor component of a mixed dish; whilst the quantities presented are lower than previously reported given that we adjust for the non-seafood component of mixed dishes.

**Figure 3:**
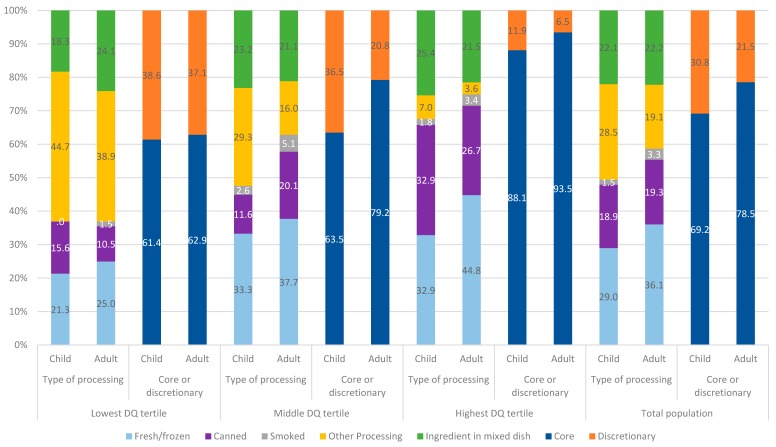
Proportion of total seafood consumed (among seafood consumers) according to type of processing and as core or discretionary by diet quality tertiles. DQ = diet quality.

Figure 3 shows the proportion of seafood consumed by adults and children by the type of processing and whether the seafood consumed was as a core food or discretionary food, according to different levels of overall diet quality. Approximately 21 and 31% of total seafood consumed by adults and children respectively is consumed as a discretionary food. In other words, the seafood has been prepared or processed in a way that it contains considerable added fats insofar that it no longer contributes to ‘core’ foods recommended for consumption in the ADGs. As would be expected, the proportion of seafood consumed as a discretionary food increases as overall diet quality decreases. This is generally consistent with previous analysis showing the consumption of lower quality seafood by consumers of lower socioeconomic status [41], which is also associated with lower overall diet quality [54]. Among seafood consumers, children across all levels of diet quality consumed a greater proportion of seafood as discretionary food compared to adults. Regardless of diet quality ranking, children reported consuming similar total quantities of seafood (*p* = 0.072), whereas adults in the lowest diet quality tertile consumed significantly less than those in the middle and highest tertile of diet quality (*p* < 0.001). The reported consumption of canned seafood was greater for children (*p* = 0.002) and adults (*p* < 0.001) in the highest diet quality tertile, whilst processed seafood was much lower (*p* < 0.001), compared to those in the lowest diet quality tertile. Adults reporting the highest diet quality also reported consuming a higher quantity of fresh or frozen seafood compared to adults reporting lowest diet quality (*p* < 0.001) (see Appendix A Table A1). The quantity of seafood from mixed dishes (children *p* = 0.391, adults *p* = 0.795) and for children fresh or frozen seafood (*p* = 0.153), reported intakes were similar across all groups (Figure 3 and Appendix A Table A1). 

### 3.4. Sustainability

A comparison of selected sustainability considerations related to fisheries and aquaculture food systems and products used in Australia is presented in Table 1. Sustainability assessments across organisations are focussed on the production side of seafood supply, in particular on the production of the target species in wild capture fisheries. The broader ecological impacts of fishing do not form part of formal government assessments but can be assessed as part of a fisheries’ ecological risk assessment process and managed under individual workplans. There can, however, be considerable variation in how ecosystem and habitat impacts are assessed, and this does not necessarily reflect whether an assessment includes specific consideration of ecosystem structure and function. Economic sustainability is not formally assessed by any organisations, although the Australian Government does evaluate the economic performance of fisheries against the economic objective of the Fisheries Management Act 1991. Labour rights is the only social sustainability consideration to be formally assessed. The ASC also notes some social considerations as part of their social impact assessment. Assessment of global-scale abiotic resource use and emission-based environmental impacts, such as global warming, toxicity and eutrophication, is absent, except for the ASC’s inclusion of eutrophication. Resource use is not considered by the Australian Government, but some aspects of resource use are examined by the other organisations. For example, the AMCS assesses the amount of fishmeal used in aquaculture but not the resources used for provision of terrestrial feed ingredients such as soy or chicken by-product. Formal assessment, or even partial examination, of seafood sustainability post-harvest is lacking. Of all the considerations identified, only traceability was assessed by any organisation.

Total waste and loss of fish and seafood throughout the system was estimated at approximately 125,300 t with the largest components occurring at production prior to landing (also known as discards at sea, 32,300 t) and by consumers at the household level (26,300 t) (Figure 1). Note that these estimates provide a general guide only in the absence of empirical data. A national reporting system for bycatch (of which discards is a component) does not exist and data is relatively limited. However, recent estimates of discards from four Australian fisheries jurisdictions estimate discard rates between 14 and 58% of total catch, with an overall discard rate of 46% [55]. This is considerably higher than the 12% used here from the FAO study which may relate to the low discard rate estimates for USA’s federally managed fisheries of 17%; and suggests that the estimates presented here are underestimated. 

## 4. Discussion

In light of the growing trend in seafood consumption in Australia, this analysis of fish and seafood consumption patterns linked to production systems and sources of major species and products, highlights both opportunities and challenges for ensuring fish and seafood contribute to healthy and sustainable diets in future. These opportunities and challenges, including practical applications are described in the following paragraphs. The main limitation to this work relates the paucity of data available that characterizes the various elements of the fish and seafood food system in Australia. The most recent consumption data used, whilst of high quality in methods and detail, is only available from December 2011 and, due to cost, is not collected routinely. No information is available on when the next survey will be conducted, which limits our ability to understand constantly changing trends in consumption. Furthermore, data on the post-harvest handling and storage, processing and packaging, distribution and retail of fish and seafood is largely held by the private sector and not available in a consistent format for analysis. This reflects the current nature of food systems where the different elements often operate in relative isolation. Whilst a key strength of the analysis presented here is the triangulation of various data sources to provide a holistic systems view, this could be greatly enhanced by a whole-of-food system approach to the monitoring and evaluation of food system activities, interactions and governance.

From a nutritional perspective, the quality of different fish and seafood products varies according to species and the way in which they are processed and prepared (see Appendix A Table A2 on nutrient composition of selected fish and seafood products in Australia). Cooking tends to reduce the relative moisture content of foods, thereby increasing concentration of some nutrients, but can also reduce the content of others. For example, greater cooking times and temperatures can increase lipid oxidation and lead to decreases in fat content [56,57]. The effects of smoking on nutrient content are also variable; heat and related moisture loss can lead to nutrient losses, while certain chemicals in smoke (depending on the method of smoking) can also promote nutrient retention through prevention of oxidation, thereby reducing oxidative changes to protein, fats and vitamins [58]. Canning of fish and seafood involves heat treatment in order to eliminate contamination with pathogens, which can result in similar concentration and losses of nutrients to cooking; though mineral content tends to be relatively stable during processing involving heat treatment [59]. Of greater nutritional significance, however, are nutrient composition changes related to the addition of other food ingredients during processing and preparation. The sodium content of canned tuna (in brine) for example is approximately 400% higher than raw or baked tuna (see Appendix A Table A2). The total fat content of crumbed or battered and deep-fried white fish is several fold higher than baked or grilled white fish (see Appendix A Table A2). In this context, the relatively high proportion of seafood consumed as a discretionary food is of concern, particularly among children. Growth of processed fish and seafood consumption between 1995 and December 2011 suggests that this trend may continue [8]. Strategies that promote increased seafood consumption for health should focus on fresh, frozen, canned (preferably in water or oil) or smoked options whilst limiting the consumption of processed seafood that contributes to discretionary food intake. This also highlights a need for linking consumers with the appropriate skills and resources to prepare seafood dishes from fresh, frozen or only minimally processed seafood, rather than relying on more ‘ready-to-consume’ processed products with added ingredients. That said, given that the majority of seafood (57%) consumed is distributed via foodservice outlets as ready-to-eat seafood, consumers should not be the only target of health or nutrition initiatives. Incentives, resources and skills needed for various foodservice institutions such as schools, hospitals, catering, takeaway outlets and restaurants to serve seafood as a minimally processed core food should be explored. 

The type and level of processing of seafood products also has implications for sustainability, though this is largely understudied. Increasing levels of processing will increase the use of abiotic resource inputs, potentially increasing environmental impacts. However, this could be partially offset when processing alleviates the need for cold storage and extends shelf life (for example, in preparation of canned seafood products). LCA studies tend to focus only on the resource use of seafood production and there are few examples which compare different types of processing. One study from Australia based on environmentally extended input output analysis shows higher GHGE per unit of purchase price for frozen fish, compared to canned fish, compared to fresh fish [60]. Growing demand for processed seafood products also has implications for aspects of social sustainability, related to the growing trend of outsourcing fish processing to low- and middle-income countries, particularly in Asia, for re-import. Countries such as China and Thailand can offer highly competitive labour costs, which can offset the costs of global transport [61]. However, the maintenance of safe and appropriate working conditions remains a significant challenge in the region [62].

A notable challenge related to sustainability is that the majority of seafood consumed in Australia, and other importing countries, comes from imported sources where traceability and data on the sustainability of the source is often limited [63], though this is also an issue for domestically sourced seafood. Canned tuna (mostly imported from Thailand) and fresh or frozen prawns (mostly imported from China, Thailand and Vietnam) are two of the most commonly consumed seafood products in Australia. Thailand is the biggest canned tuna producer in the world, with the tuna being sourced mainly from, but not limited to, the Western Central Pacific Ocean. The target species, fishing methods, bycatch rates and other elements of sustainability related to canned tuna often varies by brand and product so it is difficult to comment on the relative proportion of canned tuna sold through retail channels in Australia that would be considered sustainable. Exceptions are products from capture fisheries that have MSC certification at the point of sale. These products are sourced from sustainably managed fish stocks and can be traced from capture to retail. The sustainability of imported prawns can also be difficult to assess because of issues related to traceability. Analogous to the MSC, the ASC certification scheme identifies aquaculture products and producers which meet certain environmental and social sustainability criteria which differ by species. Whilst various producers and prawn products from Australia’s major sourcing countries have the ASC certification, again, the relative sustainability for this broader category of seafood (excluding those with certification) is difficult to assess at the retail level. The AMCS recommend avoiding the consumption of imported prawns on the basis of environmental degradation of mangroves in major producing regions along with concerns regarding effluent management; though the assessments are not done on an individual farm basis and practices are likely to vary. Salmon is the most popular seafood type consumed by Australian adults, the majority of which is sourced from domestic aquaculture concentrated in Tasmania. Major salmon producers in the region have full or partial ASC certification. However, some site-specific sustainability issues remain. The AMCS recommend avoiding the consumption of farmed salmon in general related to these site-specific concerns.

An opportunity for improving sustainability of the fish and seafood food system in Australia lies in addressing waste and loss throughout the supply chain, particularly at production and consumption. It is estimated that approximately 35% of global fish catch is lost or wasted through discarding at sea or along supply chains [48], with approximately 13% of catch wasted during processing [64]. Addressing waste issues in the food system generally is complicated by a lack of food waste data and there is growing recognition that waste levels need to be quantified in order to effectively target and design waste reduction interventions [65]. This analysis estimated total waste and loss of more than 125,000 t (which is also noted to likely be an underestimate), equal to more than half of total domestic production. A key driver of waste and loss in production relates to the management of discards. Discards account for around 8% of the total global catch by volume [66], but have been reported as high as 88.7% of the catch in some fisheries [67]. A significant proportion of discarded fish are edible. Recent analysis in Australia has shown that at least 50,000 t of underutilised fish and seafood, comprising of more than 100 different species, are caught each year as bycatch [68]. Solutions to address fisheries discards have been directed at the catch sector. However, more emphasis is needed on the market and consumer end of the seafood supply chain where the fundamental challenge lies in creating markets for less familiar (often lower cost) products with unstable supply chains [69]. Encouraging consumers to eat a wider range of species, including those that are currently discarded, has been promoted as a sustainable approach to seafood consumption [70,71]. In addition to educating consumers on a wider variety of species and preparation methods, targeting foodservice operators (which accounts for the majority of seafood retail in Australia) may be more effective and appealing from a cost-saving perspective. Creating markets for a greater diversity of currently underutilised fish and seafood species also holds significant potential to alleviate pressure on overfished stocks and associated ecosystem degradation, and could support significantly greater levels of fish consumption globally in a sustainable manner [70]. A potential entry point for further expansion lies within the Asian food culture in Australia where a large diversity of fish and seafood is culturally acceptable. 

Other approaches to improve the sustainability of the fish and seafood industry include encouraging processing plants to use every part of the fish they purchase and using by-products from fish carcasses for consumption and other purposes [63]. During the processing of fish, the fillets are retained while the bulk of product (60–70%), consisting of head, viscera and frame, is typically discarded. Within the Australian seafood industry, thousands of tonnes of fish waste are produced each year by processors and retailers [72]. Some of this fish waste is rendered, but most is sent to landfill, ground-and-discarded or otherwise diverted from human consumption. Due to a lack of commercially available technology to recover proteins and lipids from fish processing by-products, this resource typically remains unavailable for human consumption [73]. Optimising the use of fish for human consumption is an important sustainability consideration. A good example is Asian seafood markets in major cities in Australia where fish head and carcasses are routinely sold as food. These processing by-products have high demand in Asian communities around the country. Other opportunities include greater recovery of flesh through mechanical deboning and the development of value-added products [74], as well as recovering functional and nutritious proteins and oil from fish processing by-products [73]. Lean manufacturing techniques can be used in processing to increase yields and reduce waste, while packaging improvements can enable fish to be stored for longer [64]. The hospitality and food service sector also generates a huge amount of food waste arising from spoilage, food preparation and consumer plates [75]. Seafood loss also varies by retailer. For example, retailers with seafood service counters typically have higher seafood loss than those selling packaged fish only. A growing number of retailers are offering prepared seafood products that generally have a longer shelf life than fresh seafood [76], though as noted in the discussion above this can have implications for nutritional quality and sustainability. A number of approaches have emerged to address consumer waste including smart fridges, mobile apps for consumers at home, improved packaging (re-sealable, prolonging shelf-life of food) and specific storage guidelines [77]. 

This analysis has also highlighted the relatively narrow conceptualisation of ‘sustainability’ in the common frameworks used to assess this aspect of the fish and seafood sector in Australia. Of the three domains of sustainability (environmental, economic and social), current frameworks overwhelmingly focus on environmental aspects and these are concentrated at the production end of the food system with little consideration at the processing, distribution, retail and consumption stages. Environmental assessments also focus on targeted species and do not consider broader impacts on ecological structure and biodiversity. This presents an opportunity for economic, social and environmental sustainability to be more holistically integrated into the fish and seafood food system from production right through to consumption. Doing so requires a whole-of-food system approach which recognises both the multiple goals of the food system, and how the various elements and activities throughout the system are connected and interact. 

## Figures and Tables

**Table 1:** Matrix comparing the different components of sustainability considered by different sustainability assessment frameworks used in Australia.

Supply Chain Stage	Sustainability Considerations	Assessment Organisations			
		Aust Govt	AMCS	ASC	MSC
Production (Fishing and farming)	Biological stock status (target species)	Y	Y	NA	Y
	Fishing effort	Y	Y	NA	Y
	Bycatch (including by-products and discards)	P	Y	NA	Y
	Ecosystem and habitat impacts	P	Y	Y	Y
	Labour rights of employees	N	N	Y	P
	Economic performance	Y	N	N	N
	Equity, livelihoods, gender	N	N	P	N
	Animal welfare	N	N	N	N
	Effectiveness of governance	N	Y	N	Y
	Use of antibiotics	N	N	Y	N
	Biosecurity (disease and invasive species)	N	N	Y	N
	GHGE	N	N	N	N
	Eco-toxicity	N	N	N	N
	Eutrophication	N	N	Y	N
	Resource use (land, water, energy, fishmeal)	N	P	P	P
	Waste/loss (not including discards)	N	N	N	N
Post-harvest handling and storage Processing and packaging Retail and distribution Consumption	Product yield, use of coproducts	N	N	N	N
	Ecosystem and habitat impacts	N	N	N	N
	Labour rights of employees	N	N	N	N
	Equity, livelihoods, gender	N	N	N	N
	Animal welfare	N	N	N	N
	Effectiveness of governance	N	N	N	N
	GHGE	N	N	N	N
	Eco-toxicity	N	N	N	N
	Eutrophication	N	N	N	N
	Resource use (land, water, energy amount/ type)	N	N	N	N
	Waste/loss	N	N	N	N
	Distance transported	N	N	N	N
	Transport mode	N	N	N	N
	Traceability	N	N	Y	Y

Y (green) = included in sustainability assessment; P (yellow) = partially examined but not included as part of sustainability assessment, or only some aspects included, for example MSC requires audits of labour practices for supply chain companies if a risk of forced or child labour practices is established; N (red) = not included in sustainability assessment; NA = not applicable; GHGE = greenhouse gas emissions; AMCS = Australian Marine Conservation Socity; ASC = Aquaculture Stewardship Council; MSC = Marine Stewardship Council.

## Appendix A

**Table A1:** Mean quantity of different categories of fish and seafood consumed by adults and children according to diet quality tertiles (g/person/day).

Seafood Category	Lowest Tertile DQ				Middle Tertile DQ				Highest Tertile DQ				Tertile Comparison		Total			
	Children		Adults		Children		Adults		Children		Adults		Children	Adults	Children		Adults	
	Mean	SD	Mean	SD	Mean	SD	Mean	SD	Mean	SD	Mean	SD	*p* value	*p* value	Mean	SD	Mean	SD
**Whole Population**																		
Total fish/seafood	10.2	33	26	65.8	12.7	40.2	32.8	77.2	14.2	40.4	35.1	79.5	0.072	<0.001 (a, b)	12	37.4	30.9	73.9
Proportion consuming any seafood (%)	12.8		22.1		16.4		26.3		16.6		27.8		0.032	<0.001 (a, b)	15		25.1	
**Seafood Consumers**																		
Total fish/seafood	79.2	54.9	117.7	93.6	77.9	69.3	125	105.8	85.5	60.8	126.5	105.9	0.602	0.177	80.3	62.3	123	101.9
**Seafood Type**																		
Salmon	4.5	26.8	12.1	42.7	10.5	32.4	19.1	53.5	15.6	41.4	34.6	78.8	0.034	<0.001 (b, c)	9.7	33.5	22	61.2
Tuna	15.4	31.6	11.8	39.1	9.2	28.5	24.6	53.6	31.2	54.4	27.9	55.6	<0.001 (b, c)	<0.001 (a, b)	17.2	38.9	21.4	50.4
Barramundi	1.4	12.9	6.6	36	2.5	20.1	3.3	25.5	1.7	11.2	4.7	30.9	0.821	0.119	1.9	15.7	4.9	31.2
Sardine	0	0	0.8	8.5	0	0	2.5	23.6	1.4	8.7	2.4	13.1	0.025	0.065	0.4	4.5	1.9	16.2
Other fish	36.9	60	49.2	85.4	38.2	57.1	45.4	86.5	26.9	48	37.3	84	0.245	0.017	34.8	56.2	43.9	85.4
Total fish	58.2	63	80.5	94	60.4	65.3	94.8	98.9	76.8	63.2	106.9	104.8	0.055	<0.001 (b)	63.9	64.4	94.1	100
Prawns	6.2	23.9	17.3	43.2	7.9	28.6	14.6	46.6	5	18.1	10.6	40.1	0.638	0.007 (b)	6.6	24.6	14.1	43.4
Other crustaceans	4.2	18	1	11.3	0	0	1.5	18.6	0	0	1.8	21.6	<0.001 (a, b)	0.63	1.5	10.9	1.4	17.7
Total crustaceans	10.4	29	18.3	44.3	7.9	28.6	16.1	50.6	5	18.1	12.4	45.1	0.282	0.037	8.1	26.5	15.6	46.7
Oysters	0	0	1.3	10.1	0	0	1.1	12.8	0	0	0	0	-	0.011	0	0	0.8	9.3
Other molluscs	2.7	16	8.7	28.4	6.8	21.3	3.4	27.6	0.6	6.3	3.1	26.8	0.009 (c)	<0.001 (a, b)	3.7	16.8	5.1	27.7
Total molluscs	2.7	16	10	29.9	6.8	21.3	4.5	30.3	0.6	6.3	3.1	26.8	0.009 (c)	<0.001 (a, b)	3.7	16.8	5.9	29.2
Unspecified seafood	7.9	22.7	8.9	37.9	2.8	27.5	9.6	43.9	3.1	13.8	4.1	21.7	0.113	0.003 (c)	4.7	23	7.5	35.6
**Level of Processing**																		
Fresh/frozen, cooked (otherwise unprocessed)	16.9	45.9	29.4	67.1	25.9	50	47.1	99.7	28.1	54.2	56.7	100.2	0.153	<0.001 (a, b)	23.3	50	44.4	90.9
Canned	12.4	28.9	12.4	41.5	9.1	28.2	25.1	56.5	28.1	50.7	33.8	58.6	<0.001 (b, c)	<0.001 (a, b, c)	15.2	36.5	23.8	53.4
Smoked	0	1.3	1.7	12.3	2.1	14.5	6.4	31.2	1.6	11.8	4.3	23.7	0.254	<0.001 (a)	1.2	10.9	4.1	23.6
Other Processed	35.4	57.5	45.8	84.2	22.8	42	20	52.7	6	20.8	4.6	22.8	<0.001 (b, c)	<0.001 (a, b, c)	22.9	45.8	23.5	61.4
Unspecified/ingredient in mixed dish	14.5	26.7	28.3	54.4	18	49.8	26.4	51.4	21.7	42.8	27.2	62.2	0.391	0.795	17.7	41.1	27.3	56.3
**Core or Discretionary Fish/Seafood**																		
Core	43.7	49.1	68.4	82.5	53.6	70.2	101.4	107.2	79	64.5	121.3	106.7	<0.001 (b, c)	<0.001 (a, b, c)	56.7	63.5	97	101.7
Discretionary	35.4	57.5	49.2	85.5	24.3	43.4	23.6	57.4	6.5	21.4	5.2	25	<0.001 (b, c)	<0.001 (a, b, c)	23.6	46.3	26	63.9

a = sig dif between lowest tertile and middle tertile (*p* < 0.01); b = sig dif between lowest tertile and highest tertile (*p* < 0.01); c = sig dif between middle tertile and highest tertile (*p* < 0.01).

**Table A2:** Nutrient composition of selected fresh and processed fish and seafood products in Australia.

Food Name	Eng	Mois	Pro	Fat	Tot SFA	Trans FA	Tot MUFA	Tot PUFA	ω-3 FA	Ca	Fe	Zn	I	Se	Na	Vit A RAE	Vit B12	DFE	Vit D3 eq	Vit E
	kJ	g	g	g	%	%	%	%	mg	mg	mg	mg	ug	ug	mg	ug	ug	ug	ug	mg
Barramundi, fillet, raw	523	73.8	19.2	5.3	32.21	3.1	38.59	29.25	543.0	19	0.32	0.36	2.3	29.4	56	19	1.9	0	10.7	2.61
Barramundi, fillet, grilled, no added fat	717	64.1	26.4	7.3					743.9	25	0.43	0.5	3	40.2	76	24	2.3	0	13.19	3.58
Flathead, flesh, raw	395	77.7	21.1	1	39.6	2	25.4	34.6	188.3	63	0.2	0.6	0	42	97	0	1.8	0	2.1	0.14
Flathead, flesh, baked, no added fat	541	69.5	28.9	1.4					257.9	86	0.27	0.82	0	57.5	133	0	2.2	0	2.59	0.19
Mackerel, raw	561	71.7	19.3	6.3	35.86	0.1	30.02	34.12	1604.2	11	0.44	0.49	29	36.5	59	39	2.4	1	2.1	0.69
Mackerel, grilled, no added fat	768	61.2	26.4	8.6					2197.5	15	0.6	0.67	37.7	50	81	45	2.7	1	2.3	0.94
Salmon, Atlantic, fillet, raw	965	62.3	20.5	16.7	25.14	3.2	47.11	28.62	2192.9	48	0.31	0.42	0	23.2	32	10	2.2	0	6	2.49
Salmon, Atlantic, fillet, grilled, no added fat	1078	57.8	22.9	18.6					2450.1	54	0.35	0.47	0	26	36	9	2	0	5.36	2.78
Sardine, Australian, whole, raw	442	72.9	19.7	2.9	45.25	0.2	28.49	28.92	612.4	725	3.95	3.1	75.8	98	665	106	8.3	7	6	0.46
Sardine, Australian, whole, fried, no added fat	444	70.5	22	1.9					337.2	873	6.1	3.29	52	97.5	794	91	6.8	10	5.65	0.44
Snapper, fillet, raw	405	77	20.3	1.6	41.4	2	28.9	29.4	265.4	123	0.3	0.7	40	35	85	5	2.5	0	2.1	0.6
Snapper, fillet, baked, no added fat	554	68.6	27.8	2.2					363.6	168	0.41	0.96	52.1	47.9	116	6	3.1	0	2.59	0.82
Tuna, yellowfin, flesh, raw	435	71	23.4	1	34.07	2	23.1	42.67	243.0	16	0.73	0.52	28	37	37	18	0.5	2	2.1	0.5
Tuna, yellowfin, flesh, baked, no added fat	596	60.3	32.1	1.4					332.8	22	1	0.71	36.4	50.7	51	21	0.6	2	2.3	0.68
Whiting, King George, flesh, raw	362	77.6	19.8	0.7	37.1	2	27.5	36.1	111.2	37	0.2	0.8	10	55	70	0	1.5	0	2.1	0.56
Whiting, King George, flesh, baked, no added fat	517	67.9	28.2	1					158.9	53	0.29	1.14	13.6	78.6	100	0	1.9	0	2.7	0.8
Prawn, flesh, raw (green)	381	76.8	20.7	0.8					137.4	72	0.16	1.43	57.2	43.7	345	2	1	16	0	1.59
Prawn, flesh, cooked from raw, no added fat	507	68.6	27.1	1.2					176.1	185	0.93	2.19	30.9	40.1	1077	1	1.4	24	0	1.03
Squid or calamari, raw	328	81	16.7	1.2	43.3	2	7.1	49.7	333.4	11	1.3	1.3	20	44.8	284	1	1.3	5	2.1	1.2
Squid or calamari, fried, no added fat	410	76.2	20.9	1.5					416.7	14	1.62	1.62	23.8	56	355	1	1.4	5	2.36	1.5
Fish finger, crumbed, purchased frozen, grilled, no added fat	1050	51.5	13.8	13.2					756.1	15	0.51	0.46	4.8	27.3	341	15	0.5	74	0.61	0.51
Fish, white flesh, battered, packaged frozen, baked, no added fat	820	55	12.2	7	11.6	2	59.1	26.7	119.7	21	0.5	0.4	10.7	29	383	1	0.5	1	0.3	3.22
Fish, battered or crumbed, from takeaway outlet, deep fried, blended frying fat, ready to eat	1006	51.1	15.2	13.2	36.41	0.8	36.1	26.92	32.3	21	0.5	1	1	29	406	1	1	5	0.3	0.21
Shark, battered, deep-fried, takeaway outlet	1090	54.2	15.5	14.5	24.59	2	43.46	29.64	80.0	11	0.4	0.38	3.4	27.3	295	12	0.2	0	0.3	3.1
Anchovy, canned in oil, drained	762	49.5	25.4	8.9	23.3	3.2	42.7	34	769.4	167	2.5	2.9	13	68.1	5480	4	0.9	13	6.36	3.33
Cod, smoked, raw	356	81.1	18.8	1	35.2	2	35.5	29.2	179.2	20	0.2	0.3	50	27	655	7	0.3	0	2.1	0.41
Cod or hake, smoked, steamed	397	76.6	20.1	1.5	34	2	33.7	32.5	302.4	20	0.2	0.5	0	34	548	5	0.3	0	2.2	0.46
Salmon, pink, canned in brine, drained	579	70.9	20.7	6.2	25.22	1.1	38.8	34.88	1659.4	191	1.14	1.05	60	34.2	382	16	1.3	13	10.6	0.93
Salmon, smoked, sliced	787	62.4	24.3	10.1	19.2	0.1	51.3	28.7	854.5	8	0.31	0.38	7.4	16	1015	0	1.5	0	4.2	0.6
Tuna, canned in brine, drained	540	74.4	26.1	2.6	44	0.6	21.3	34	751.9	5	1.16	0.76	10.2	72.6	200	0	1.1	26	2.7	0
